# Investigation on Ge_0.8_Si_0.2_-Selective Atomic Layer Wet-Etching of Ge for Vertical Gate-All-Around Nanodevice

**DOI:** 10.3390/nano11061408

**Published:** 2021-05-26

**Authors:** Lu Xie, Huilong Zhu, Yongkui Zhang, Xuezheng Ai, Junjie Li, Guilei Wang, Anyan Du, Zhenzhen Kong, Qi Wang, Shunshun Lu, Chen Li, Yangyang Li, Weixing Huang, Henry H. Radamson

**Affiliations:** 1 Key Laboratory of Microelectronics Devices & Integrated Technology, Institute of Microelectronics, Chinese Academy of Sciences, Beijing 100029, China; xielu@ime.ac.cn (L.X.); zhangyongkui@ime.ac.cn (Y.Z.); aixuezheng@ime.ac.cn (X.A.); lijunjie@ime.ac.cn (J.L.); duanyan@ime.ac.cn (A.D.); kongzhenzhen@ime.ac.cn (Z.K.); wangqi@ime.ac.cn (Q.W.); lushunshun@ime.ac.cn (S.L.); lichen2017@ime.ac.cn (C.L.); liyangyang@ime.ac.cn (Y.L.); huangweixing@ime.ac.cn (W.H.); 2 Microelectronics Institute, University of Chinese Academy of Sciences, Beijing 100049, China; 3 Research and Development Center of Optoelectronic Hybrid IC, Guangdong Greater Bay Area Institute of Integrated Circuit and System, Guangdong 510535, China

**Keywords:** vertical Gate-all-around (vGAA), p^+^-Ge_0.8_Si_0.2_/Ge stack, dual-selective wet etching, atomic layer etching (ALE)

## Abstract

For the formation of nano-scale Ge channels in vertical Gate-all-around field-effect transistors (vGAAFETs), the selective isotropic etching of Ge selective to Ge_0.8_Si_0.2_ was considered. In this work, a dual-selective atomic layer etching (ALE), including Ge_0.8_Si_0.2_-selective etching of Ge and crystal-orientation selectivity of Ge oxidation, has been developed to control the etch rate and the size of the Ge nanowires. The ALE of Ge in p^+^-Ge_0.8_Si_0.2_/Ge stacks with 70% HNO_3_ as oxidizer and deionized (DI) water as oxide-removal was investigated in detail. The saturated relative etched amount per cycle (REPC) and selectivity at different HNO_3_ temperatures between Ge and p^+^-Ge_0.8_Si_0.2_ were obtained. In p^+^-Ge_0.8_Si_0.2_/Ge stacks with (110) sidewalls, the REPC of Ge was 3.1 nm and the saturated etching selectivity was 6.5 at HNO_3_ temperature of 20 °C. The etch rate and the selectivity were affected by HNO_3_ temperatures. As the HNO_3_ temperature decreased to 10 °C, the REPC of Ge was decreased to 2 nm and the selectivity remained at about 7.4. Finally, the application of ALE in the formation of Ge nanowires in vGAAFETs was demonstrated where the preliminary I_d_–V_ds_ output characteristic curves of Ge vGAAFET were provided.

## 1. Introduction

As the continuous scaling down of complementary metal-oxide-semiconductor (CMOS) technology nodes, novel device designs and high-mobility channel materials have been under investigation [1,2,3,4,5,6,7,8]. Vertical nanowire GAAFET is a powerful candidate for the 3 nm process, since its superiority in the short channel effects (SCEs) control [9,10] and can greatly reduce the gate pitch and increase the device integration density [11,12]. In addition, Ge is one of the most promising channel materials for pMOS due to its high carrier mobility and excellent bandgap [13]. Therefore, vertical GAAFETs with Ge as channel material have become an ideal choice for next era CMOS technology.

**Figure 1:**
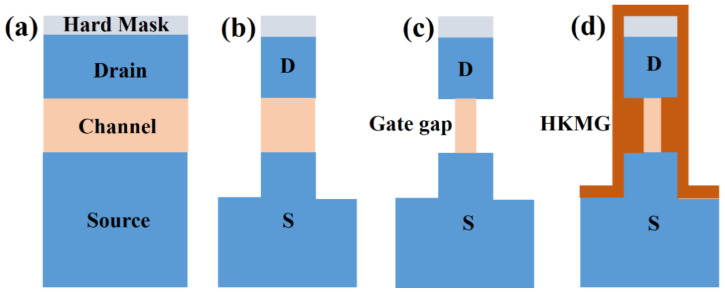
Schematic diagram of the basic flow of vertical Gate-all-around FET (vGAAFETs). (**a**) Sandwich structure and hard mask growth, (**b**) lithographic patterning and plasma anisotropic etching, (**c**) channel isotropic selective etching to form laterally depressed channels and gate gaps, (**d**) gate gaps filling with high-k metal gate (HKMG).

For vertical GAA devices, a new structure of vertical sandwich GAAFETs (VSAFETs) with Si source/drain and SiGe channel has been proposed [14,15]. The main process flow of VSAFETs is shown in Figure 1, the selective etching of the channel is a key step in the formation of vertical nanostructures. And the dimension of the channel is determined by selective etching. For the formation of Ge vertical nanowires, several selective etching methods have been reported, including dry etching with Cl_2_ or CF_4_ RF plasma [16,17,18] or mixtures of Cl_2_/HBr [19] and wet etching with H_2_O_2_ (HNO_3_) [20] or TMAH [21] or other alkaline solutions [22] or mixtures of HF/H_2_O_2_/CH_3_COOH [20,23]. SiGe/Ge multilayer structures have been used to release with SiGe sacrificial layer etching to fabricate Ge nanowires in the lateral GAA device [19,24]. However, the above-mentioned methods for forming Ge nanowires are all continuous etching methods, and the etching depth is time-dependent. They achieve high selectivity and high etch rate at the cost of repeatability. At and beyond the 3 nm technology node, the gate, and channel of vGAAFETs need to be precisely aligned and the size controlled at the atomic-scale to achieve good device performance. In order to form Ge channels with self-aligned gate structure in VSAFETs, Ge was required to be laterally released to nano-scale size with source/drain-selective etching of Ge. Atomic layer etching (ALE) [25,26,27] is a promising technology that can remove ultra-thin materials through at least one self-limiting reaction step to achieve lower atomic-scale process variation. At present, the ALE for isotropic selective etching of SiGe to Si [28,29,30] has been reported, ALE for isotropic selective etching of Ge to GeSi has not been extensively reported. However, the ALE is not universal, and the selective ALE method depends on the recipe and it is difficult to achieve.

In this work, a developed wet dual-selective ALE process with selective etching of Ge and crystal-orientation selectivity of Ge oxidation was proposed. Based on the principle of atomic layer etching (ALE) and the oxidation–removal reaction of HNO_3_ and deionized (DI) water, the characteristics of Ge_0.8_Si_0.2_-selective ALE of Ge at different temperatures were investigated systematically. The ALE process with a focus on the selective etching of Ge in p^+^-Ge_0.8_Si_0.2_/Ge multilayers with 70% HNO_3_ as oxidizer and DI water as oxide removal. The saturated relative etched amount per cycle (REPC) and selectivity at different HNO_3_ temperatures between Ge and p^+^-Ge_0.8_Si_0.2_ were investigated in detail. The application of ALE in the formation of Ge nanowires in VSAFETs was demonstrated.

## 2. Materials and Methods

The samples were performed on 200-mm p-type Si (100) wafers with a resistivity of 8–12 Ohm·cm. The high-quality epitaxial p^+^-Ge_0.8_Si_0.2_/Ge vertical heterostructure multilayers started with a Ge buffer layer growth by ASM E2000 (ASM, Munich, Germany) plus RPCVD on Si wafers [20]. Dichlorosilane (SiH_2_Cl_2_), germane (10% GeH_4_ in H_2_), and diborane (1% B_2_H_6_ in H_2_) were utilized as gas precursors for Si, Ge, and B, respectively. Ge_0.8_Si_0.2_ layers were in-situ doped with boron (concentration: 1.0 × 10^19^ cm^−3^). The growth parameters and boron content have been carefully optimized to avoid boron precipitates in the Ge_0.8_Si_0.2_ layers [31]. The Ge buffer layer was grown with a two-step growth of low-high temperature (400 °C and 650 °C), and then a post-growth in situ annealing was applied at 820 °C in H_2_ ambient [32]. Then, the p^+^-Ge_0.8_Si_0.2_/Ge stacks were grown at 500 °C using an adjusted gas source with H_2_ as a carrier gas. Then, a hard mask with 30 nm SiN and 50 nm SiO_2_ was deposited with plasma enhanced chemical vapor deposition (PECVD) on the epitaxial p^+^-Ge_0.8_Si_0.2_/Ge stack layers. Finally, the p^+^-Ge_0.8_Si_0.2_/Ge stack fins were patterned by I-line optical lithography and fabricated by using HBr-based dry anisotropic etching. Afterward, the samples were cut into small slices to facilitate the etching experiments.

**Figure 2:**
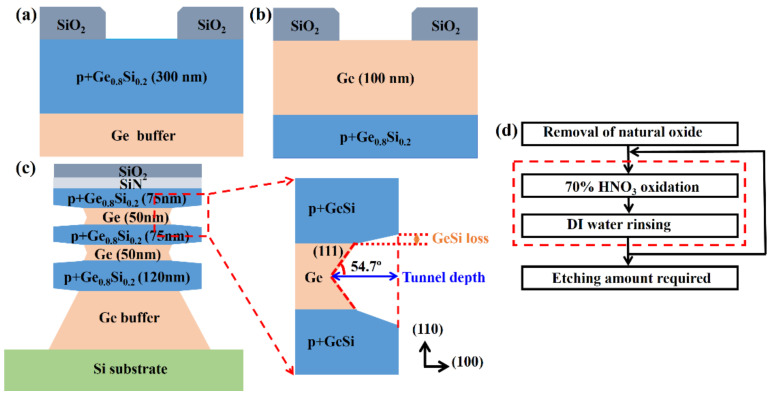
(**a**) Scheme of structure for p^+^-Ge_0.8_Si_0.2_ (**a**) and Ge (**b**) with (100) flat surface; (**c**) scheme of p^+^-Ge_0.8_Si_0.2_/Ge stacks with SiO_2_ and SiN as hard mask. The tunnel depth and GeSi loss are indicated in the insert view, selective etching of Ge at (111) planes result in a lateral angle of 54.7°. (**d**) Flow diagram of HNO_3_-DI water for p^+^-Ge_0.8_Si_0.2_-selective etching of Ge with ALE.

There were three kinds of samples to estimate the etch rate and selectivity between Ge and p^+^-Ge_0.8_Si_0.2_, as shown in Figure 2a–c. The etch rate of Ge (100 nm) and p^+^-Ge_0.8_Si_0.2_ (300 nm) films with (100) flat surfaces were expressed by the etch rate per cycle (EPC). In order to measure the relative etch rate and selectivity of Ge and p^+^-Ge_0.8_Si_0.2_ with ALE, a structure with (110) sidewall was fabricated and keep p^+^-Ge_0.8_Si_0.2_/Ge/p^+^-Ge_0.8_Si_0.2_/Ge/p^+^-Ge_0.8_Si_0.2_ at 120 nm/50 nm/75 nm/50 nm/75 nm, and p^+^-Ge_0.8_Si_0.2_ with boron dopant concentration of 1.0 × 10^19^ cm^−3^. And the thickness and composition of the samples were kept constant among repetitive experiments. The prepared p^+^-Ge_0.8_Si_0.2_/Ge stack structure is shown in Figure 2c. The relative total etched amount (tunnel depth) and GeSi loss are shown in the insert view, selective etching of Ge at (111) planes result in a lateral angle of 54.7°. The etch selectivity between p^+^-Ge_0.8_Si_0.2_/Ge stacks with (110) sidewall is estimated by (tunnel depth + GeSi loss)/GeSi loss [28]. The relative etched amount per cycle (REPC) was calculated as the tunnel depth divided by the number of etching cycles. The flow diagram of ALE is shown in Figure 2d, the steps in the dashed frame are one cycle of ALE, including 70% HNO_3_ oxidation, deionized (DI) water rinsing, and repeating the number of cycles until the required etching amount was reached. Before etching experiments, the samples were immersed in diluted BOE (dBOE, 49 wt% HF and 40 wt% NH_4_F with volume ratio of 1:7) for 5 min to remove the natural oxide. During the experiments, the volume of the nitric acid solution was constant at 2 L, and the DI water was with overflow rinsing. As high-concentration nitric acid is easier to decompose, the experimental process requires a high-precision density meter to monitor the concentration of HNO_3_ solution. In view of the different oxidation mechanism of nitric acid (HNO_3_) concentration on Ge, the nitric acid concentration must be maintained at 70% with 5% variation in the ALE experiments compared to the continuous etching of Ge with nitric acid at low concentration. The experimental temperature was kept at 20 °C ± 0.5 °C, and the temperature with variation ±0.5 °C of the HNO_3_ solution was controlled by the water bath method of the cryostat at low temperature. The oxidation time t_ox_ of the control recipe is 30 s and the DI water rinsing time (oxide remove time) is set as 1 min to make sure that oxide is removed totally.

Scanning electron microscopy (SEM) was used to examine the morphology and etching depth of the samples. Transmission electron microscopy (TEM) characterized the sample to determine the layer profile and evaluate the results of wet etching. Energy dispersive spectroscopy (EDS) was employed to determine the elemental analysis of the etched layers. High-resolution X-ray diffraction (HRXRD) was used to measure the strain relaxation and examine the epitaxial quality. Atomic force microscopy (AFM) measured surface roughness.

## 3. Results and Discussion

### 3.1. Dual-Selective Etching Ge to p^+^-Ge_0.8_Si_0.2_ with ALE

**Figure 3:**
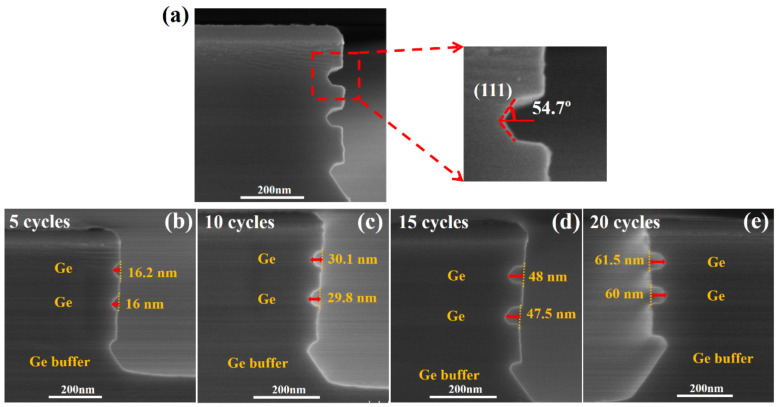
(**a**) The dual-selective etch of Ge at (111) planes result in a lateral angle of 54.7°. The SEM cross-section images of p^+^-Ge_0.8_Si_0.2_/Ge multilayers after 70% HNO_3_-DI water ALE at (**b**) 5 cycles, (**c**) 10 cycles, (**d**) 15 cycles, and (**e**) 20 cycles. (Oxidation time t_ox_ = 30 s; temperature of HNO_3_ = 20 °C).

In order to form Ge channels with self-aligned gate structure in pVSAFETs, Ge was required to be laterally released to nano-scale size with ALE. First, we tried the ALE recipe of selective SiGe to Si in the previous work [28,29], using H_2_O_2_ and diluted HNO_3_ solution as oxidants to oxidize p^+^-Ge_0.8_Si_0.2_/Ge stacks. However, due to the water-soluble of GeO_2_, GeO_2_ generated by the reaction of Ge with H_2_O_2_ and diluted HNO_3_ solution was dissolved in water immediately, resulting in uncontrolled continuous etching [20], these recipes cannot be used as ALE recipes for Ge. Then, we used O_2_ plasma and O_3_ as oxidants, combined with the etchant for Ge selective etching experiments, but failed with non-selectivity (not shown). Finally, we found that (1) only a high concentration of HNO_3_ (70% with 5% variation) as oxidant and DI water as etchant can achieve the selective ALE of Ge to p^+^-Ge_0.8_Si_0.2_, and (2) 70% concentrated HNO_3_ has crystal-orientation selectivity for the oxidation of Ge, but not for p^+^-Ge_0.8_Si_0.2_. The oxidation rate of HNO_3_ on Ge surface is inversely proportional to the atomic density of the crystallographic plane, which determines the slower oxidation rate on Ge (111) planes [33,34]. Therefore, the self-saturated selective etching of Ge at (111) planes result in a lateral angle of 54.7°, as shown in the insert view of Figure 3a. Within the experimental error, this angle is equal to the theoretical angle between the (100) and (111) set of planes. A developed dual-selective ALE method, including material selectivity and crystal-orientation selective oxidation, is suitable for the selective etching of Ge in p^+^-Ge_0.8_Si_0.2_/Ge stacks.

Due to the ALE separated into two parts: oxidation and oxide remover. The amount of etching is determined according to the amount of oxidation, so the oxidation step is the key to control the etching rate. First, 70% HNO_3_ was used to periodically oxidize the surface of the sample for time t_ox_, showing a self-limiting surface passivation reaction, and then deionized (DI) water was used to directly remove the oxide. The oxidation of Ge in high-concentration nitric acid can be described as follows [20]:

(1) $$\mathrm{Ge}+4{\mathrm{HNO}}_{3}\to {\mathrm{GeO}}_{2}\cdot H_{2}O+4{\mathrm{NO}}_{2}↑+{\;H}_{2}O$$

In the reaction of high-concentration nitric acid to germanium, the reaction product is GeO_2_∙H_2_O. In the whole reaction process, GeO_2_ formed at the germanium-liquid interface diffuses slowly, and enough GeO_2_ covers the entire germanium surface and produces passivation to prevent further corrosion of germanium [35]. Compared with ALE of SiGe to Si, the ALE of Ge to p^+^-Ge_0.8_Si_0.2_ did not need another etchant (hydrofluoric acid or dBOE) but only DI water can directly remove oxides. To ensure the stability of the nitric acid concentration during the ALE experiment, the nitric acid concentration needs to be monitored with a high-precision density meter every 5 min. It was found that the concentration of HNO_3_ changes by 5% within 1 h during the ALE experiment. When the nitric acid concentration changed more than 5%, that is, the nitric acid concentration is less than 65% (original nitric acid concentration is 70%), the p^+^-Ge_0.8_Si_0.2_/Ge stacks became continuous etching in HNO_3_ solution. In order to maintain the stability and repeatability of the ALE experiment, the nitric acid solution was changed every 1 h. It can be seen that the concentration of nitric acid had a great influence on the experimental results. Since nitric acid is exothermic and volatilized when exposed to water, the sample must be dried before the next cycle in nitric acid. In order to verify whether germanium oxide is completely removed in DI water, three comparative experiments were taken at the same time: (1) 70% HNO_3_ 30 s + DI water cleaning for 1 min, (2) 70% HNO_3_ 30 s + DI water cleaning for 2 min and (3) 70% HNO_3_ 30 s + DI water cleaning for 1 min + dBOE immersing for 1 min + DI water cleaning for 1 min. Excluding measurement errors, the Ge etching amounts of these three cleaning conditions were almost the same (not shown), which proved that 1 min of DI water cleaning was sufficient to remove germanium oxide without adding other etching agents. It was also proved that enough water molecules in DI water can pass through GeO_2_ to reach the Ge-GeO_2_ interface and strip the germanium oxide.

Samples were immersed in concentrated HNO_3_ (70%) with oxidation time t_ox_ = 30 s at HNO_3_ temperature of 20 °C, and rinsed in DI water for at least 1 min to remove GeO_x_ effectively. Figure 3b–e shows the SEM cross-section images of p^+^-Ge_0.8_Si_0.2_/Ge stacks after etching with 5 cycles, 10 cycles, 15 cycles, and 20 cycles, respectively. Within the measurement error, the mean relative etching amounts of Ge in p^+^-Ge_0.8_Si_0.2_/Ge stacks were 16.1 nm, 30 nm, 47.8 nm, and 60.8 nm, respectively. The relative etching amount is linearly proportional to the number of etching cycles, and the etch rate per cycle (EPC) is independent of the etching cycle number with the mean value of 3.1 nm.

In p^+^-Ge_0.8_Si_0.2_/Ge stacks, the selective ALE of Ge can be performed smoothly in a 70% HNO_3_-DI water system. One is that the Ge layers (atoms) were oxidized preferentially by HNO_3_ owing to the weaker Ge-Ge bond energy than those of Ge-Si and Si-Si [36,37,38,39]. Second, GeO_2_ generated in the Ge layer is soluble in water, while SiO_2_ generated by oxidation of the p^+^-Ge_0.8_Si_0.2_ layer is stable and hydrophobic, and GeO_2_ far away from the surface cannot be directly dissolved in water because of the passivation of the SiO_2_ layer.

**Figure 4:**
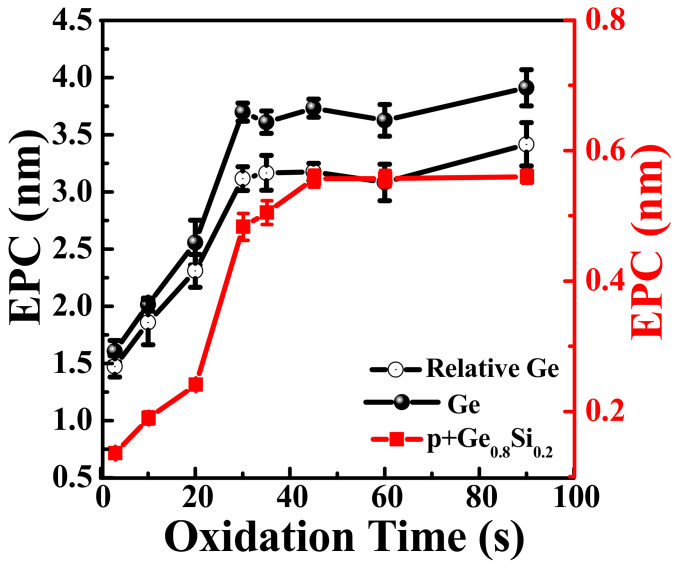
EPCs of ALE with different oxidation time for Ge and p^+^-Ge_0.8_Si_0.2_ (100) planes and Ge selectively etched in p^+^-Ge_0.8_Si_0.2_/Ge stacks. (30 cycles; Temperature of HNO_3_ = 20 °C; Experimental data of average values and error bars are shown in the graph).

The EPC was calculated by dividing the relative etching amounts by the number of etching cycles and this experiment was carried out at HNO_3_ temperature of 20 °C. The EPC for Ge and p^+^-Ge_0.8_Si_0.2_ as a function of the oxidation time is shown in Figure 4. The EPC increased with the increase of oxidation time and gradually saturates when the oxidation time exceeds 30 s, indicating that the oxidation of Ge and p^+^-Ge_0.8_Si_0.2_ in concentrated HNO_3_ (70%) is quasi-self-limiting. When the oxidation time is 30 s, the etching selectivity between Ge and p^+^-Ge_0.8_Si_0.2_ in (100) flat surface is 6.5, as determined from the two oxidation curves in Figure 4. The EPC of Ge and p^+^-Ge_0.8_Si_0.2_ were 3.7 nm and 0.5 nm, respectively. The etch rate of lateral selective etching of Ge on p^+^-Ge_0.8_Si_0.2_/Ge stacks with (110) sidewall, which is more important in vertical device fabrication, is defined as the relative etched amount per cycle (REPC). Similarly, as shown in Figure 4, the REPC of Ge in the vertical structure of p^+^-Ge_0.8_Si_0.2_/Ge stacks is fixed at ~3.1 nm. The estimated data in these experiments in Figure 4 show very small etching errors. Therefore, it can be verified that the ALE method is repeatable if the measurement error is taken out.

### 3.2. Effect of HNO_3_ Temperature on Ge ALE

**Figure 5:**
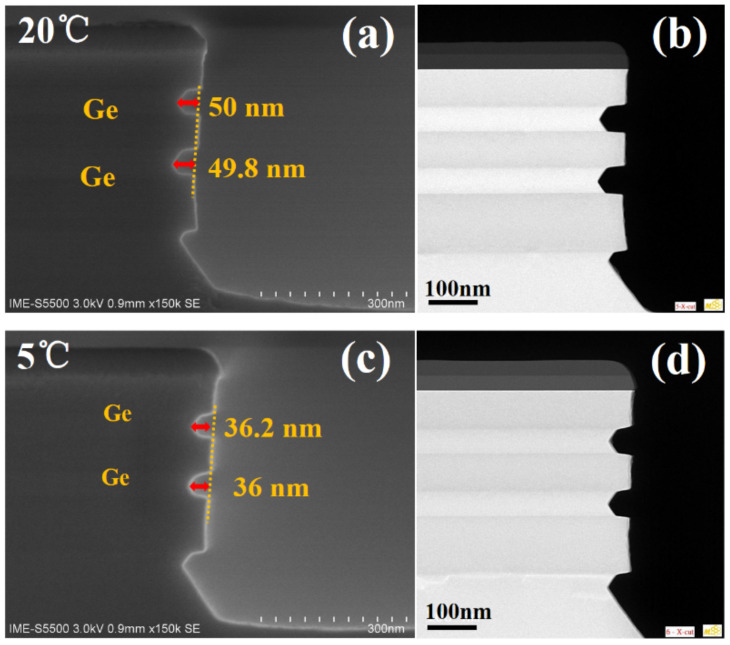
The SEM and TEM images of etching profile of p^+^-Ge_0.8_Si_0.2_/Ge multilayers after ALE at (**a**,**b**) 15 cycles (temperature of HNO_3_ = 20 °C); (**c**,**d**) 20 cycles (temperature of HNO_3_ = 5 °C). The oxidation time t_ox_ = 30 s.

At room temperature (20 °C), the REPC of Ge (3.1 nm) is about 8 times higher than that of REPC of SiGe (0.4 nm) [29]. The etching rate is relatively fast. Since oxidation plays an important role in ALE, the effect of lowering the temperature of the nitric acid solution on the oxidation rate was studied to obtain a lower oxidation rate. Due to the strong corrosiveness of nitric acid, the temperature of the nitric acid solution is cooled by the water bath of the cryostat. In order to explore the effect of low-temperature on the REPC and selectivity between Ge and p^+^-Ge_0.8_Si_0.2_, experiments were carried out at HNO_3_ temperature of 5 °C, 10 °C, 15 °C, and 20 °C, respectively. The low-temperature etching morphology was characterized by SEM and TEM. As shown in Figure 5, Ge ALE at 20 °C and 5 °C both exhibit dual-selectivity (material and crystal-orientation selectivity). The TEM results show the clear layering of the p^+^-Ge_0.8_Si_0.2_/Ge stacks and the etching morphology corresponding to the SEM characterization. The oxidation time was t_ox_ = 30 s, where the cycles of 20 °C and 5 °C were 15 cycles and 20 cycles, respectively, and the etching amount of Ge is 50 nm and 36 nm, respectively. Experiments have proved that low temperature can reduce the oxidation rate of nitric acid without changing the etching morphology, which is an effective method to reduce REPC of Ge.

**Figure 6:**
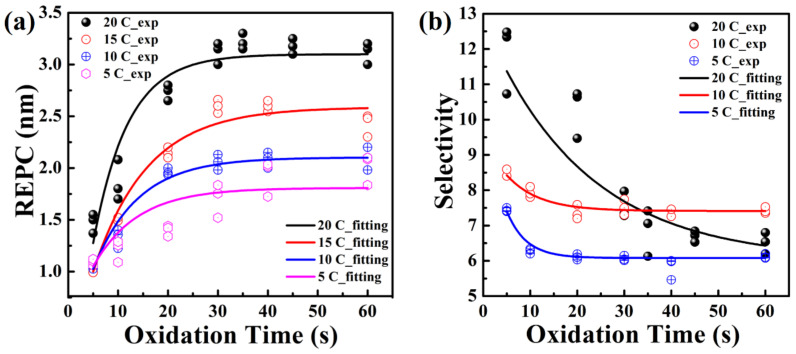
(**a**) The REPC of p^+^-Ge_0.8_Si_0.2_/Ge at different HNO_3_ temperatures (20 °C, 15 °C, 10 °C, 5 °C) as a function of oxidation time. (**b**) The selectivity between p^+^-Ge_0.8_Si_0.2_ and Ge at different HNO_3_ temperatures (20 °C, 10 °C, and 5 °C) as a function of oxidation time. The scatters and solid lines represent the experimental data and fitting curves of the experimental data.

The REPC curves of ALE at different HNO_3_ temperatures (20 °C, 15 °C, 10 °C, and 5 °C) in p^+^-Ge_0.8_Si_0.2_/Ge multilayers are shown in Figure 6a. As the temperature of HNO_3_ decreased from 20 °C to 5 °C, REPC decreased from 3.1 nm to 1.8 nm. With the HNO_3_ temperature reducing to 10 °C, the REPC of Ge was reduced to 2 nm. Similarly, the REPCs at all four temperatures reached saturation when the oxidation time exceeded 30 s, which is the quasi-self-limiting process. Due to the temperature sensitivity of the Ge oxidation reaction, the saturated oxide will be thinner with the lower HNO_3_ temperature. The temperature with variation ±0.5 °C was controlled by the water bath of the low-constant temp tank. According to the corrosion model proposed by Seidei [40], its point of view was to attribute chemical reactions to differences in energy. When the temperature of the HNO_3_ decreased, the energy difference between its molecular kinetic energy and the surface activation energy of the sample became smaller, so the oxidation rate became slower.

Figure 6b shows the etching selectivity of p^+^-Ge_0.8_Si_0.2_/Ge stacks at different HNO_3_ temperatures (5 °C, 10 °C, and 20 °C). The selectivity is defined as [28]:

(2) $$\mathrm{Selectivity}=\frac{\mathrm{GeSi}\;\mathrm{loss}+\mathrm{Tunel}\;\mathrm{depth}}{\mathrm{GeSi}\;\mathrm{loss}}$$

where GeSi loss is vertical etching amount of GeSi, which is equal to the horizontal etching amount, and the tunnel depth is the relative total etched amount of Ge to Ge_0.8_Si_0.2_, as shown in Figure 2c. The experimental data were obtained by measuring the SEM images of the etching profile in the p^+^-Ge_0.8_Si_0.2_/Ge stacks with (110) sidewalls. The mean values of p^+^-Ge_0.8_Si_0.2_/Ge selectivity at HNO_3_ temperatures of 20 °C, 10 °C, and 5 °C were 6.5, 7.4, and 6.1, respectively. The results show that Ge ALE can achieve a high selectivity of Ge to Ge_0.8_Si_0.2_, independent of HNO_3_ temperature. At HNO_3_ temperatures of 20 °C, when the oxidation time increased, there was enough time for HNO_3_ to destroy and oxidize the Si-Si bonds and Ge-Si bonds. After 30 cycles of ALE, the etching amount of Ge_0.8_Si_0.2_ became larger, and the EPC became larger accordingly. Thereby reducing the selectivity of Ge to Ge_0.8_Si_0.2_, and reached saturation when the oxidation time was 45 s. At low temperatures, due to the decrease of the molecular kinetic energy of HNO_3_, the selectivity of Ge to Ge_0.8_Si_0.2_ does not change much with time and reached saturation in about 20 s of oxidation time.

### 3.3. Structure Characterization and Material Quality Analysis

**Figure 7:**
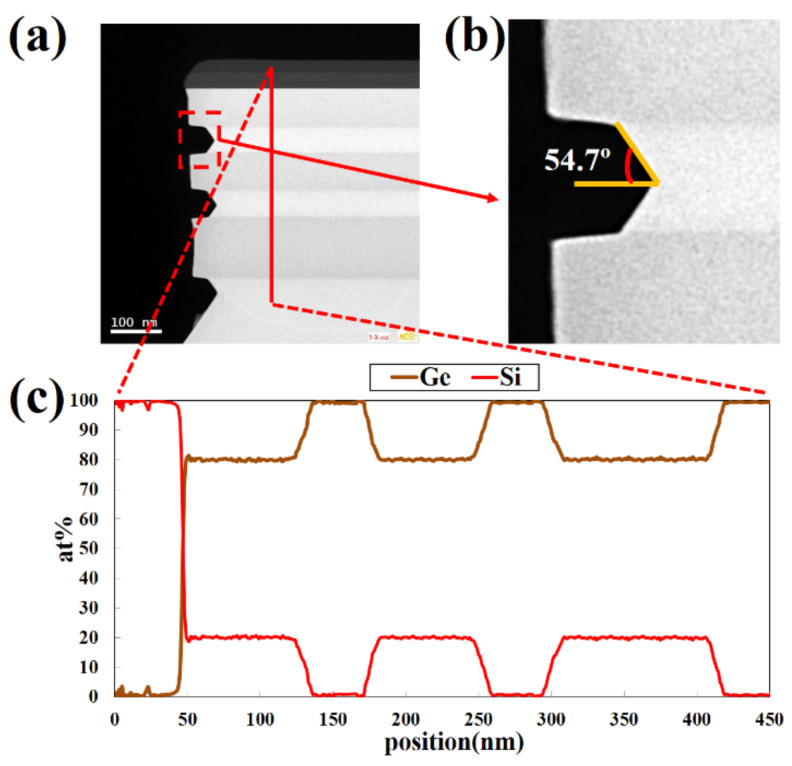
(**a**,**b**) TEM images of etching profile, and (**c**) EDS analysis with line scanning of Si and Ge in vertical orientation of the Ge buffer/p^+^-Ge_0.8_Si_0.2_/Ge stack structure. The selective etching of Ge at (111) planes results in a lateral angle of 54.7°. (20 cycles; temperature of HNO_3_ = 15 °C; oxidation time t_ox_ = 30 s).

**Figure 8:**
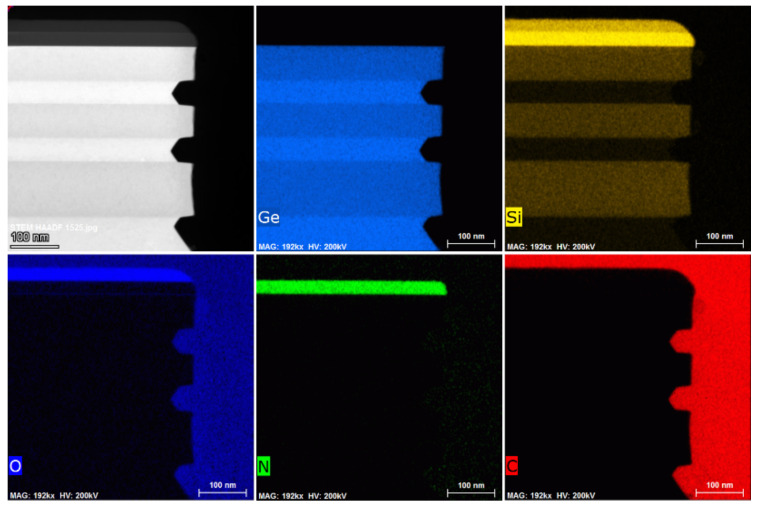
EDS mapping near etching regions with elements Ge, Si, O, N, and C, the sample etching for 15 cycles (oxidation time t_ox_ = 30 s; temperature of HNO_3_ = 20 °C).

In order to more accurately characterize the Ge ALE results in this study, the quality of the epitaxial layers and the etching morphology and element analysis of the p^+^-Ge_0.8_Si_0.2_/Ge stack structure were characterized by HRTEM and EDS. Figure 7 shows the cross-section TEM micrograph and elemental analysis of the etched area of p^+^-Ge_0.8_Si_0.2_/Ge stacks with (110) sidewalls with Ge ALE. The results show that the Ge content in the GeSi layer maintains at 80%, and there is no mixing between the layers. The relative etching amount is the same as that characterized by SEM. It was further verified that the selective etching of Ge on (111) planes resulted in a lateral angle of 54.7°. The EDS mapping of Figure 8 shows that the boundaries of the layers are obvious, the thickness of the film is consistent with the design, and there is no obvious element diffusion between the layers. The germanium content of the Ge layer is almost 100%. Si element is distributed in the layers of SiO_2_, SIN, and Ge_0.8_Si_0.2_, O mainly exists in the hard mask of SiO_2_.

**Figure 9:**
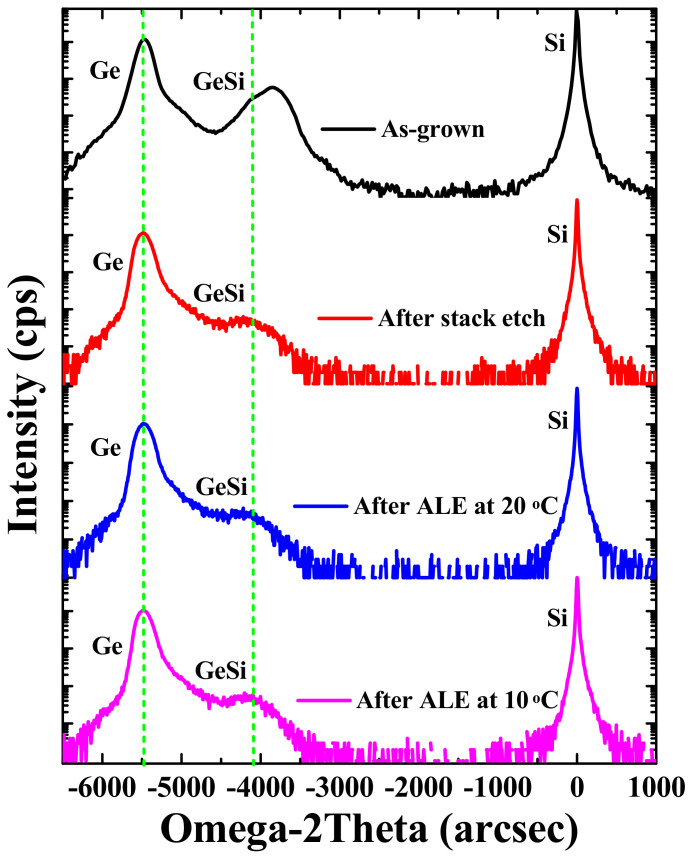
HRXRD rocking curves (RCs) around (004) reflection of stack samples of as-grown, after vertical stack etch, after ALE at 20 °C, and after ALE at 10 °C with 20 cycles.

Crystallinity and strain have a strong impact on channel carrier mobility and device performance, therefore, crystal quality and strain relaxation of the sample before and after the etching steps has been studied. HRXRD is a technique that is widely used for the detection of defects in crystal materials [41]. Figure 9 displays rocking curves (RCs) measured around the (004) reflection on stack samples: as-grown, after vertical stack etch, after ALE at 20 °C, and after ALE at 10 °C. For the as-grown sample, the Ge_0.8_Si_0.2_ and Ge peaks were intense with low Full-width-of-half-Maximum (FWHM) showing good crystalline quality of Ge_0.8_Si_0.2_/Ge stack. Since most of the film layer was removed by etching, the intensity of the Ge_0.8_Si_0.2_ peak was weaker, while the Ge peak was still strong due to the presence of the Ge buffer layer. Compared with the as-grown sample, the Ge_0.8_Si_0.2_ peak shifts towards the Ge peak after stack etching. This indicates that (tensile-strained) Ge_0.8_Si_0.2_ is partially relaxed after the vertical etching. Moreover, the Ge peak became asymmetric after vertical etch and the amount of strain in the Ge was minor. No further shift of Ge_0.8_Si_0.2_ or Ge peak was detected after ALE etching, which indicates that there was no further strain relaxation after lateral etching at 20 °C and 10 °C.

**Figure 10:**
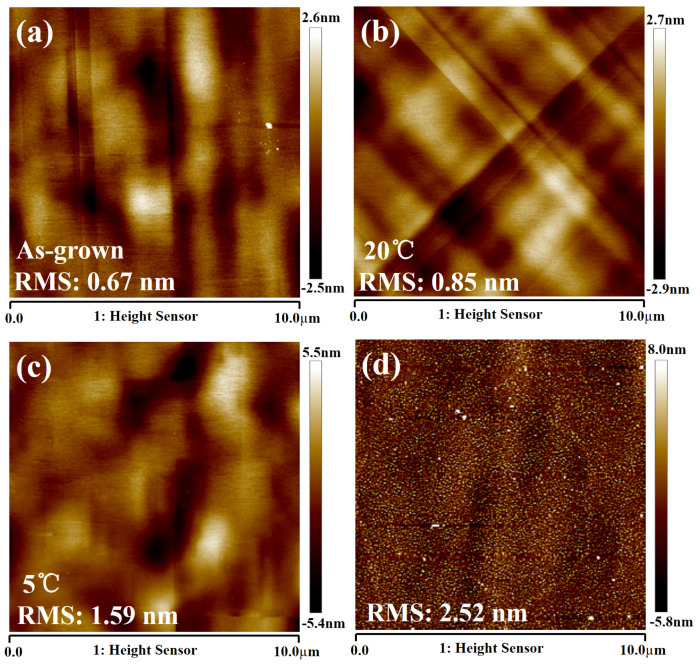
Typical AFM images (10 × 10 µm^2^) of flat (100) Ge surfaces before and after the etching process: (**a**) as–grown; (**b**) ALE with 20 cycles (temperature of HNO_3_ = 20 °C); (**c**) ALE with 20 cycles (temperature of HNO_3_ = 5 °C); (**d**) HF:HNO_3_:CH_3_COOH mixtures.

Since Ge will be used as the channel material in vertical GAAFETs, the etched germanium surface can be used as the channel interface. Due to the scattering of the surface roughness, the channel surface roughness will cause gate oxide integrity degradation and mobility degradation. Therefore, it is necessary to measure the surface roughness of Ge that has been etched many times by DI water. Figure 10 shows the AFM images of the flat (100) Ge surface with as-grown epi-Ge, after etching with ALE at HNO_3_ temperatures of 20 °C (20 cycles), after etching with ALE at HNO_3_ temperatures of 5 °C (20 cycles), and after etching with HF:HNO_3_:CH_3_COOH mixtures. It was found that the root mean square (RMS) roughness of the ALE process at 20 °C HNO_3_ temperatures is 0.85 nm, which is similar to that of the as-grown sample (RMS of 0.67 nm). However, the RMS increases as the temperature of HNO_3_ decreases. The roughness is very poor etching with HF:HNO_3_:CH_3_COOH mixtures. Table 1 shows the comparison of the RMS of flat (100) Ge surfaces of as-grown and different etching processes. It is demonstrated that the surface roughness of ALE is better than chemical continuous etching. The smoothing effect can be explained by a model, where the depressions on the surface asperity are preferentially oxidized, and the protrusions on the asperity are preferentially etched. When the temperature of nitric acid decreases, the oxidizing ability decreases, resulting in uneven surface oxidation. Finally, the height difference between the depressions and the protrusions was increased during the etching, so the surface after the low-temperature treatment is rougher. In summary, we need to make a trade-off between the etching rate and the surface roughness and choose a suitable temperature for ALE etching. Because of the equipment, the preparation process of our devices was mainly carried out at room temperature.

### 3.4. Application of ALE for Ge Vertical Sandwich GAAFETs (VSAFETs)

**Figure 11:**
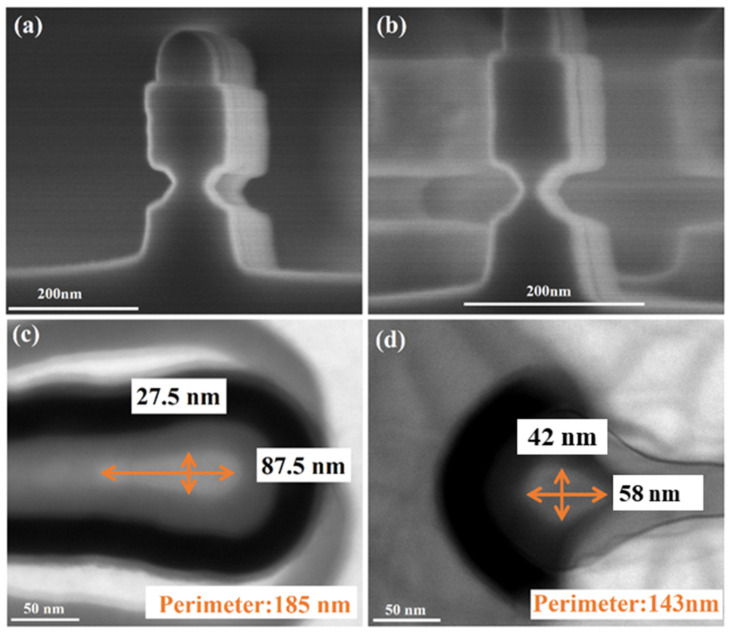
Cross-sectional SEM images of Ge selective etching with ALE, (**a**) 40 nm Ge channel with 15 cycles of ALE, and (**b**) 15 nm Ge channel with 20 cycles of ALE. Cross-sectional TEM top views of Ge VSAFETs: (**c**) NS with perimeter 185 nm, (**d**) NW with a square cross-section with perimeter 143 nm. Oxidation time t_ox_ = 30 s and temperature of HNO_3_ = 20 °C.

The ALE of Ge selective to p^+^-Ge_0.8_Si_0.2_ will be adopted in the vertical sandwich GAAFETs (VSAFETs) to form Ge channel nanowire, as mentioned in the introduction. The cross-sectional SEM images of the p^+^-Ge_0.8_Si_0.2_/Ge/p^+^-Ge_0.8_Si_0.2_ sandwich structure forming vertical nanowire are shown in Figure 11a,b. Figure 11a,b respectively show a 40 nm Ge channel with 15 cycles of ALE and a 15 nm Ge channel with 20 cycles of ALE, implying the well-controlled Ge channel size with ALE. Figure 11c,d show the TEM top view for NS with perimeter 185 nm and NW with perimeter 143 nm formed by 30 cycles of ALE, respectively. The TEM top views were cut at the top drain, the brighter part is the channel, and the black part is metal. The vertical nanosheet (NS) and nanowire (NW) shown in Figure 11c,d were obtained on the same wafer, and the channel size and shape are determined by initial dimensions (defined by electron-beam lithography) and the dual-selective ALE.

**Figure 12:**
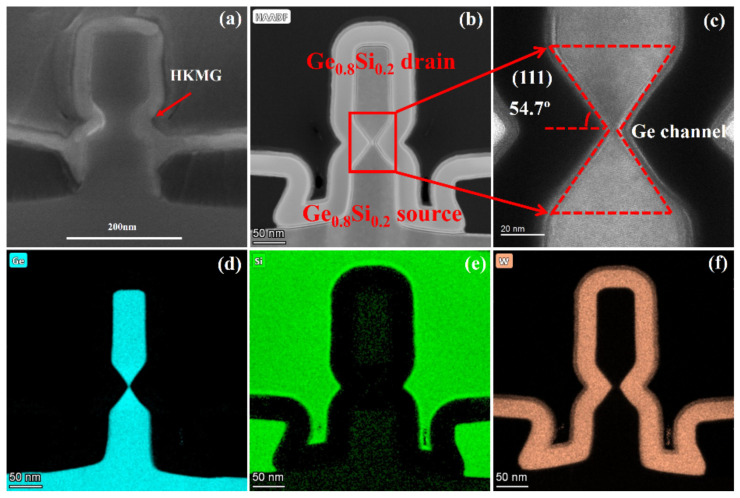
(**a**) SEM and (**b**) TEM images of Ge pVSAFET with gate gaps filling high-k metal gate (HKMG). (**c**) The self-saturated dual-selective etch of Ge at (111) planes result in an hourglass shape with a lateral angle of 54.7°. Energy-dispersive Xray spectroscopy (EDX) mapping of (**d**) Ge (cyan), (**e**) Si (cyan), and (**f**) W (yellow) atoms with sharp contours.

Figure 12a,b show the cross-sectional SEM and TEM images of the filled high-k metal gate (HKMG) of the gate gap formed by selective etching of the channel with ALE, respectively. Figure 12c shows the TEM image of the gate stack on the side-wall of the hourglass-shaped Ge channel. As shown in Figure 12d–f, the EDX mapping of elements Ge, Si, and W shows sharp contours, proving the absence of element intermixing. The self-saturated dual-selective etch of Ge at (111) planes result in an hourglass-shape with a lateral angle of 54.7°. The current transports along the (111) planes of the hourglass-shaped Ge channel.

**Figure 13:**
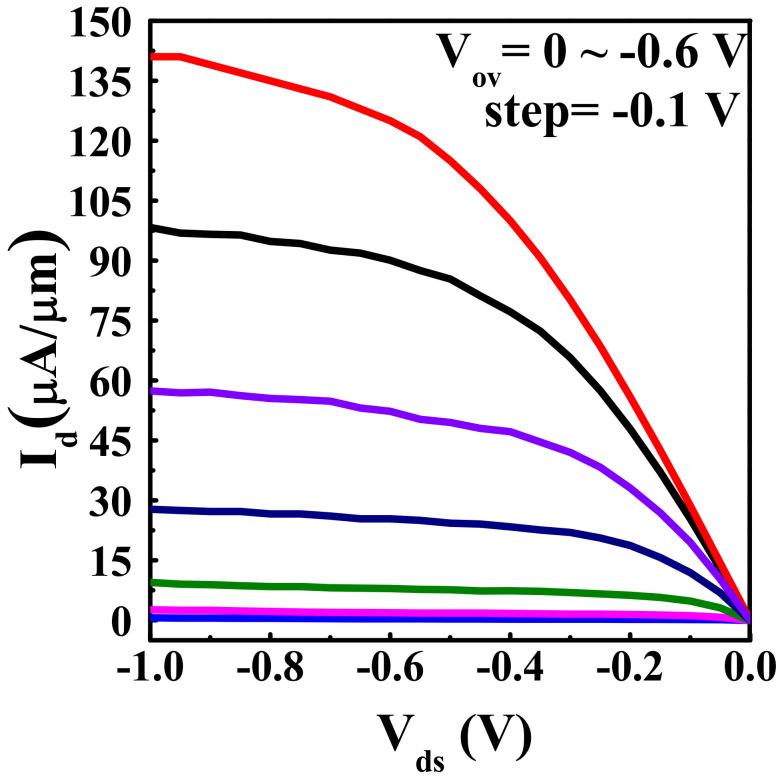
I_d_–V_ds_ output characteristic curves for Ge pVSAFET with NS thickness of ~27.5 nm, V_ov_ from 0 to −0.6 V, showing characteristic current saturation behavior. I_d_ normalizes with the perimeter of NS.

In this stage, a vertical sandwich GAAFET (VSAFET) was processed by ALE method when a Ge nanosheet (NS) with thickness of ~27.5 nm was the channel material as shown in Figure 11c. The process flow of the transistor includes sandwich structure growth, lithographic patterning and plasma anisotropic etching, channel selective etching to form channels, and filling of the high-k metal gate (HKMG) by ALD [14]. Figure 13 illustrates the I_d_–V_ds_ output characteristic curve when the I_on_ is 141 µA/um (I_d_@V_ov_ = V_gs_ − V_t_ = −0.6 V, V_ds_ = −1.0 V). It is important to emphasize that these results are preliminary for Ge pVSAFETs, and the device performance will be further studied in the future.

## 4. Conclusions

In this work, a developed wet dual-selective ALE process with selective etching of Ge and crystal-orientation selectivity of Ge oxidation was proposed. With the oxidation–removal reaction of 70% HNO_3_ and deionized (DI) water, the characteristics of Ge_0.8_Si_0.2_-selective ALE of Ge at different HNO_3_ temperatures were investigated systematically. In p^+^-Ge_0.8_Si_0.2_/Ge stacks with (110) sidewalls, the saturated relative etched amount per cycle (REPC) of Ge was 3.1 nm and the saturated etching selectivity was 6.5 at an HNO_3_ temperature of 20 °C. The etch rate and the selectivity were affected by HNO_3_ temperatures. As the HNO_3_ temperature decreased to 10 °C, the REPC of Ge was decreased to 2 nm and the selectivity remained at about 7.4. The Ge channel size in the VSAFETs was well-controlled by ALE. The hourglass-shaped channel of the VSAFETs is formed by the dual-selective ALE of Ge, narrow in the middle and wide close to S/D. Finally, the preliminary I_d_–V_ds_ output characteristic curve of Ge pVSAFET was demonstrated.

## Figures and Tables

**Table 1:** RMS of flat (100) Ge surfaces with as-grown film and different etching processes.

	As-Grown	20 °C ALE	15 °C ALE	10 °C ALE	5 °C ALE	HF:HNO_3_:CH_3_COOH
RMS (nm)	0.67	0.85	1.12	1.39	1.59	2.52

## Data Availability

The data presented in this study are available on request from the corresponding author.
